# Cancer of the Intestines

**Published:** 1910-06-11

**Authors:** E. Rock Carling

**Affiliations:** Surgeon to the Seamen's Hospital Society, Assistant Surgeon Westminster Hospital, Dean of the Westminster Hospital Medical School.


### Hospital Clinics.

### CANCER OF THE INTESTINES.

By E. Rock Carling, M.B. (Lond.), B.S., F.R.C.S., Surgeon to the Seamen's Hospital Society, Assistant Surgeon Westminster Hospital, Dean of the Westminster Hospital Medical School.

(Illustrated by Lantern-slides and Specimens, and delivered at the London School of Clinical Medicine, Greenwich).

In selecting the subject of cancer of the intestines for a clinical lecture, the important consideration is not any wealth of new light thrown upon the question by recent observations or discoveries. On the contrary, the criteria for early diagnosis are still quite inadequate, and by their very meagreness tend to be forgotten or neglected. It is, therefore, important constantly to refresh the memory as to what is actually known.

For statistical purposes the term " cancer " may be restricted for the moment to carcinoma. Combining the figures for several pathological institutes, it is found that about 10 per cent. of all necropsies are for carcinoma and about 10 per cent. of these for carcinoma of the intestine. The figures amongst patients in hospital are higher. Thus, of 762 patients with carcinoma in the Westminster Hospital, no fewer than 150 had the disease in the intestine, *i.e.* roughly 20 per cent.

Sarcoma of the intestine is so much rarer that the figures are not quite comparable. Nothnagel states that of 274 cases of sarcoma only 12, or less than 5 per cent., were primary in the intestine. Of cancer of the intestine, the rectum is answerable for about 60 per cent.; in the whole small intestine, from the duodenum to the end of the ileum, less than 5 per cent. are found. Of 128 cases in the colon, Lanz states that 24 were in the cæcum, 10 in the ascending colon, 9 at the hepatic and 17 at the splenic flexures, 15 in the transverse, 4 in the descending, and 49 in the sigmoid colon. The occurrence of both carcinoma and sarcoma actually at the ileo-cæcal valve, which I shall indicate to you by specimens, is fairly common. The differences in incidence as to sex are not very important, but, on the whole, males predominate.

As regards age, carcinoma most commonly occurs between 40 and 60, whilst sarcoma is most frequent between 20 and 40; but there are plenty of instances of carcinoma much earlier than 40, and, on the other hand, Madelung mentions a patient of 52 with sarcoma, and Smoler, one at 70. I shall show you a specimen of carcinoma of the highest part of the rectum which occurred in a boy of 11. Garrard reported a case in the sigmoid in a child of 12, Czerny one at the age of 13, and Lanz one at 21. Nothnagel mentions cases at 3½ and 3 years. Ablon collected 10 cases of sarcoma of the intestine in children. The oldest patient I have operated upon for cancer of intestine was over 90.

The object of quoting these dry figures is to point the fact that cancer of the intestines, even apart from that of the rectum, is of considerable frequency. Both the importance and the difficulty of early diagnosis can be emphasised by the observation that the majority are first recognised only when symptoms of obstruction, acute or sub-acute, supervene. In twelve months I have operated on seven such cases, and the results, for reasons which I shall indicate presently, were bad. When it is remembered that in favourable circumstances the operative mortality of resection of the colon may be as low as 12 per cent., and that the outlook as to permanent cure after deliberate operation upon anatomical principles is, for cancer, very good, it cannot but impress one with the urgent need for careful attention to everything likely to aid in early recognition of the disease.

Carcinoma of the intestines is almost always of the columnar-celled type. Occasionally the cells are polyhedral and not suggestive of an origin from the columnar mucosal cells. Colloid and myxomatous changes are of common occurrence. Sarcomata are generally of the round-celled or lymphomatous type, but mixed-celled and spindle-celled specimens have been reported.

### Incidence of Carcinoma.

The figures already given sufficiently indicate the predilection of carcinoma for the rectum and the flexures. Incidence at the flexures has been a little over-emphasised by the desire to indicate occurrence where traumatic irritation may be supposed to be at a maximum. Sarcoma is seldom found in the colon except at the ileo-cæcal valve, but is indifferently distributed over the small intestine and the rectum. Carcinoma exhibits a general tendency to obstruct the lumen of the gut, frequently constricting all coats so that it appears as if a string had been tied tightly round the outside, or converting the walls into a stiff, thick-walled tube two or three inches in length, or, again, encroaching upon the mucous surface as a proliferating, tuberous, or papilliferous mass. Whilst all these forms are common in carcinoma, in sarcoma it is seldom that the passage is narrowed. A rounded mass may project beneath the mucosa, but more often the lumen is widened at the site of growth.

Whilst a sarcoma rarely invades the mucosa, but spreads through the muscular coats from the submucosa, stopping short only of the peritoneal covering, carcinoma, on the other hand, begins in the mucosa, and though often spreading only between the coats for a long time, eventually extends into the serosa and involves surrounding tissues, adjacent organs, or neighbouring coils of gut. Whereas it is the rule for the internal surface of a carcinoma to be ulcerated, often very extensively, as in the rectum, it is the exception for ulceration to occur in sarcoma; when present, ulcers are usually small, though they may suffice for infection of the general peritoneal cavity.

Secondary carcinomata and sarcomata are found in the intestine; they may be true metastases or merely extensions, as for example from carcinoma of the uterus or from the head of the pancreas. Metastatic deposits are often button-like nodules upon the mucosa, but may have the annular extent which is characteristic of the primary type.

Metastases from primary carcinomata are, as a rule, of late appearance, and are for long limited to the regional lymphatic glands; but in the late stages it is common to find them in the liver, more rarely in the lungs and kidneys. It is notable that such deposits are of even slower growth than the original tumour, an important point in connection with excision. The work of Jamieson and Dobson has to some extent diminished the confidence with which we were wont to dogmatise about the infrequency and lateness of glandular involvement, but they have done a good deal by their anatomical demonstrations to facilitate correct operative technique.

Sarcomata involve regional glands early and extensively, and give rise to widely diffused secondary deposits in the viscera; all sorts of pressure effects may arise in the late stages. In the distended portion of the gut immediately above the constriction, fæces tend to accumulate; partly as a result of the friction of hard masses compressed by hypertrophied muscle and partly as the outcome of putrefaction, ulcers form above the growth, generally close, but sometimes, as Coupland and Morris showed, as far away as the cæcum, even when the growth was in the rectum. Fistulæ, communicating with other coils of gut or with the exterior, localised abscesses, and even general peritonitis are in consequence not uncommon. Although as a rule the section of intestine just below the growth is contracted, and the walls very thin by comparison with the greatly hypertrophied and infiltrated gut above, yet this is not always the case. In the specimen of jejunal cancer I shall show the gut below was dilated. In this instance it led to a search for a secondary cause of obstruction, the more so because in no fewer than six of the 70 known cases there has been more than one primary growth in the small intestine. Both carcinoma and sarcoma at times give rise to massive tumours outside the gut, filling the pelvis or bulking largely in the abdomen. When the peritoneum is reached, great masses may be formed or the whole surface may be sown with minute nodules, the omentum and mesentery being extensively involved. Ascites, turbid, milky, or bloody, is then a clinical feature of the case.

A malignant tumour of the gut may give rise to intussusception and to volvulus, acute obstruction supervening. Acute distension of the colon or cæcum occasionally arises; for the explanation of this phenomenon we are indebted to the late Mr. Barnard.

These anatomical and pathological considerations are, however, only a necessary preliminary to the consideration this afternoon of the clinical facts upon which diagnosis may be based.

It very rarely happens that any symptoms are recognisable before some degree of stenosis is established. The period that elapses between the initiation of the growth and encroachment upon the lumen varies greatly. It may extend, in the type that depends upon contraction of the connective tissue matrix, over as much as two or three years. In the exuberant type that fills the lumen with its own bulk, the time may be but a matter of weeks.

### Facts for Diagnosis.

*The anamnesis* is of the greatest importance. Not only has it bearing upon the possibility of other causes of stenosis, such as old attacks of tuberculous or other peritonitis, of appendicitis, of dysentery, or of syphilis, or of a strangulated hernia which may have left a stricture of the gut; not only such positive evidence, but negative evidence is also valuable. Due weight must be given to the statement of a patient that he has "never had a day's illness in his life," or "never been to a doctor." Trousseau long ago described the "vague dyspepsia" due to organic disease in the intestines which such patients often have as their earliest symptom. Slight abdominal discomfort or "fulness" becoming abdominal uneasiness or unrest; slight loss of appetite becoming marked and more marked until anorexia amounts to a profound distaste for food, are very suggestive. Pallor is so insidious in development, so slow in its progress, that it is often unnoticed by those about the patient, and may be regarded as habitual when attention is directed to it. The complexion loses its freshness, the skin its healthy qualities, and may even exhibit a liability to eruptions of eczematous type. Loss of strength, loss of habitual energy, a tendency to tire soon and easily, depression, taciturnity, and especially a slow but steady loss of weight, are common events in the story which attentive inquiry may elicit. On the other hand, there may be complete latency for very long periods, or at the most a mild chronic indigestion to which no importance is attached. Such latency is of longer duration when the growth is in the small intestine, or when it is low down in the large, of small size, and not ulcerated. Loss of weight is most rapid when stenosis is established high up in the small gut, on account of the disturbance of nutrition. A moderate pyrexia is not uncommon.

*Subjective phenomena* are capricious even when stenosis is reached. Nausea recurs intermittently without much reference to food. Pain may be absolutely wanting, or may be steady in its continuance and its site; where the small intestine is involved, or the ileo-cæcal region, it sometimes has definite reference to meals. In some instances it may be evoked by violent physical exertion. It is described as of a gnawing character, and, of course, when extension of the growth has involved nerve trunks, may be of severe neuralgic type. Colic varies progressively in intensity from a mild griping to agonising cramp, the latter generally determined by purgatives.

*Constipation* appearing for the first time in a patient past 40 is in itself very significant, but even in those who have been occasionally or habitually constipated the progressive character of the difficulty in getting a good action of the bowel calls for attention. Purgatives have an irregular effect.

They give relief at first, but later only after very great pain. Tenesmus is common and may be very severe, but it occurs only in cases in which the growth is in the sigmoid or rectum. It is very well known that this constipation is apt to alternate with diarrhœa. The French speak of constipation varied by intestinal "debâcles," meaning sudden fluid evacuations almost without notice. When the growth is low and the surface ulcerating, there may be "morning diarrhœa," which is merely "the voidance of accumulated discharges." It must be borne in mind that a common result of fæcal stasis is colitis, so that there may be persistent looseness of the bowels. Sooner or later there will be definite attacks of obstruction. The only common causes of chronic obstruction after forty are carcinoma and fæcal accumulation. When acute obstruction has supervened, it is rarely possible, except in the case of rectal growths, to make a diagnosis of a neoplastic origin. It is stated that the collapse is not so marked as in cases due to such a condition as strangulation by a band.

*Rectal examination* may not only reveal the actual growth if it be in the lowest part of the bowel, but affords other evidence. Ballooning of the rectum, usually associated with a spasmodically contracted sphincter, is not diagnostic of growth, but it spells organic obstruction. It may be possible to feel distended and tense coils of gut; or, if the obstruction is high up, the collapsed coils below fall into the pelvis and may be recognised.

*The stools* may be normal in appearance wherever the growth is situated; if quite low down in the rectum, or low enough in the sigmoid to give rise to spasm of the sphincter, the shape and size of the stools may be altered characteristically, but it is rarely observed. Lanz has remarked upon the persistent fetor of the evacuations, but it is seldom enough to attract the patient's attention. The presence of "slime," or mucus, is, however, often noticed. Search should be made for mucus, for pus, and for cancer cells; blood is to be observed in about 20 to 25 per cent. of cases. It has recently been asserted that "occult" blood is an early and consistent diagnostic sign of ulceration in the intestinal tract. It must be remembered, however, that the finding of corpuscles by the microscope, or the detection of blood pigment by the spectroscope, is inconclusive unless the diet has been hæmoglobin-free for a day or two. In a case of cancer of the jejunum upon which I operated, there had been stools of peculiar pultaceous character for weeks before, and the normal appearances were not observed for months after removal of the tumour.

*Inspection* of the abdomen affords a good deal of evidence. In the first place, there may be signs of loss of flesh. It must not be imagined, however, that adiposity is incompatible with cancer of the bowel; on the contrary, it may be of extreme degree. I have on three or four occasions operated through two or three inches of fat in the abdominal wall. One may mention the condition described as "acanthosis nigricans," and thought by some to be indicative of abdominal cancer. Pigmented spots, flat warts, and telangeiectases in the skin of the abdomen should be noted and the duration ascertained; but the evidence is of little value. Distension of the abdomen supervenes at some stage in almost every case. In some degree, and as a recurrent, persistent, or progressive state, it is commonly noted by the patient. It may be general, or it may be limited to the colonic area where the obstruction is low, whilst in stenosis at or above the ileo-cæcal valve it tends to be limited to the lower central zone. In very high cases distension may be absent or so restricted to the epigastric and upper umbilical regions as to be mistaken for dilatation of the stomach. This has happened twice to me, once in simple and once in malignant stricture of the jejunum, and it has been observed in other similar cases. Circumscribed meteorism or localised distension is now and then a valuable localising sign. With stricture of the hepatic flexures the cæcum alone may be blown up; with stasis at the transverse colon, Nothnagel's sign, hyper-resonance in the right costo-vertebral angle, may be found.

Visible peristalsis is a valuable sign of organic obstruction. With duodenal narrowing the wave of contraction may be observed in the stomach; with cæcal stenosis the "ladder-pattern" in the lower central region is very striking; the parallel ridges are horizontal or oblique, the individual ridges are not more than one to three inches wide, and the wave is brisk. With sigmoid obstruction the horseshoe of the colon may stand out as a wide rigid tube. One patient who had a splenic-flexure growth used to describe the contracture of his transverse colon as his "cat's back." In association with this peristalsis, audible gurglings, rumblings, and prolonged bubblings are frequently noted. They may be so loud as to be heard in the next room, and a patient has complained to me that they kept her awake. When the contents of the intestine are fluid, the noises may have a definite metallic quality, which Wilms has thought characteristic.

*Palpation* is sometimes valuable. About 40 per cent. of cases have a palpable tumour. The characters are variable. When the part affected is fixed the tumour is immobile; when arising in a section of gut with a long mesentery the tumour may be extraordinarily mobile. The site has some relation to the part in which it arises, *e.g.* small-gut tumours usually lie below and to the left of the umbilicus. The surface is generally firm or hard, and may be nodular, but the characters are complicated by the presence of fæcal masses above the obstruction, and sometimes by adherent coils of gut. A fæcal mass is generally not tender, and may be indented by steady pressure; such accumulations are very rare, apart from organic obstruction, anywhere above the sigmoid except in the cæcum itself. When observed, after thorough purgation and washing out of the lower bowel great changes will be found, and one must be careful not to overlook the growth in one's satisfaction at proving the correctness of a diagnosis of fæcal accumulation. It has been remarked that it is easier to mistake a malignant mass for fæces than the reverse. Some tumours are so small that even when the open abdomen is explored skilled fingers may miss them.

The contracture of the segment of gut above the obstruction gives a striking sensation to the examining hand, described by the Germans as "darmsteiffung," or gut-stiffening. It may, however, be observed apart from organic narrowing. Of three patients without mechanical stenosis in whom I have felt this stiffening, two had myxœdema. In malignant cases Lanz has pointed out that it may be elicited by light percussion with the fingers. The percussion note over a cancer of the intestine is generally imperfectly resonant. When the intestines are much dilated and filled with fluid fæces a pseudo-ascites may be present which is very misleading, but it differs from free fluid in that succussion is generally obtainable.

*Vomiting* is almost always a feature of some stage in the progress of the disease. The attacks of colic are often associated with nausea or emesis. With high obstruction the vomit is copious, and stomach-washings contain not only bile, but pancreatic secretion enough to hydrolyse fat; that is, of course, when the stricture is below the papilla. With low site the vomit consists only of food and gastric secretions. When acute obstruction supervenes the regurgitant type and fæculent characters are seen as usual. Vomiting of fæces occurs only when there is a communication between the colon and the stomach.

With high stenosis the urine is apt to be scanty in quantity, and sometimes contains an excess of indican, but this latter occurrence is of little significance.

### Special Signs.

Of the special signs connected with cancer of the rectum I shall say nothing. It ought not to be necessary, but I fear it is, to keep on hammering away at the importance of digital examination of the rectum in every case with the smallest symptom referable to the bowel. Is there one of us who has not to confess that he has overlooked important evidence by neglect of this elementary rule of diagnosis? Every natural orifice invites investigation. There are a few accessory means of diagnosis. Radiography, with the assistance of bismuth meals, will indicate the site of obstruction when it is in the large intestine, but in the small the stricture must be very narrow to arrest the fluid contents long enough to afford valuable evidence. I can illustrate this by a case in which I excised a stricture of the duodeno-jejunal junction. The lumen admitted only a small probe and had almost certainly been present from birth, yet the first symptoms appeared at the age of 46.

It has been proposed to feed the patient on magnesium bullets as a better aid to $x$-ray elucidation.

The plan of distending the colon with air or fluid is pursued in America. A simple air-pump or soda and tartaric acid may be used. It may be remembered that the normal content of the adult large intestine is about six quarts; but distension is not without danger. Kelly's rectal tubes, and more especially the sigmoidoscope, are often valuable aids when used with proper precautions.

It must have been noticed that almost all the signs and symptoms mentioned are those simply of stasis, and not of cancer as such. The diagnosis of the cancerous nature is generally only presumptive, but it is worth mentioning that some slight assistance might be available from clinical examination of the blood; all that can be ascertained as a rule is the existence of a secondary anæmia. The value of an estimation of the anti-tryptic content of the blood is as yet not proven.

### Differential Diagnosis.

It is beyond the scope of my present purpose to go into the question of differential diagnosis, but I would draw your attention to the accumulation of reports of pseudo-neoplastic tumours of the intestine. Besides the well-known ileo-cæcal tuberculosis and other chronic granulomatous tumours there is a class of inflammatory hyperplastic tumours, coming under the title of pericolitis, sigmoiditis, or "diverticulitis," which simulates very closely the clinical history of cancer, and which is amenable to treatment by physiological rest—*i.e.* by temporary exclusion; these tumours have been responsible for many a falsified prognosis of fatality. One mistake which I made is of interest. I diagnosed obstruction, probably malignant, somewhere near the ileo-cæcal valve. At the operation I found a very thick rigid and fixed appendix closely adherent to the mesentery of the ileum and dragging upon it so as to produce a stricture. I believed my diagnosis of cancer falsified, but the microscope showed that there was a carcinoma of the appendix. That leads me to remind you that even among intestinal cancers that of the appendix is singularly benign.

### Conclusion.

I am sorry it is impossible to present you with any touchstone for the recognition of this grave and fatal malady. My object is, however, to emphasise the enormous importance of exploratory laparotomy. Any suggestion of such a symptom complex as I have indicated above must bring the patient under unremitting scrutiny. There must be no waiting to find a "lump." As soon as there are reasonable grounds for suspicion it should be put to the patient that in the present state of abdominal surgery he stands to gain infinitely more than he risks by submitting to exploratory laparotomy. I am not advocating indiscriminate "belly-ripping"; on the other hand, I wish to insist on the most careful and deliberate attention to the minutiæ of evidence, but it is heart-breaking work to meet a series of these cases only as emergency operations when even palliative measures are too often unavailing. I am well aware that in many cases it is not the fault of the doctor; he knows nothing of the patient's state until he is called in to relieve acute symptoms; but those who enjoy most fully the confidence of their patients will have the best opportunities for early diagnosis, and there is such a thing as the education of one's patients.

---

[Next week's clinic will be by Professor Raymond of Paris, on "Clinical Forms of Tabes."]

